# Zirconia Facts and Perspectives for Biomaterials in Dental Implantology

**DOI:** 10.7759/cureus.46828

**Published:** 2023-10-11

**Authors:** Prachi V Singh, Amit Reche, Priyanka Paul, Shivangi Agarwal

**Affiliations:** 1 Public Health Dentistry, Sharad Pawar Dental College and Hospital, Datta Meghe Institute of Higher Education and Research, Wardha, IND

**Keywords:** titanium, bacterial colonization, periointegration, osseointegration, biocompatibility, zirconia

## Abstract

Dental implantology has witnessed remarkable advancements in recent years, and zirconia has emerged as a prominent biomaterial for dental implant applications. This review explores the multifaceted aspects of zirconia, focusing on its properties, processing methods, biocompatibility, mechanical performance, and clinical applications. Over the past few decades, the most popular choice of material for dental implantology has been titanium which has been found to have the highest success rate of implant treatment. However, recently, it has been observed that zirconia might replace titanium and eventually emerge as one of the gold-standard materials of dental implants. Analysis of biomechanical sciences and biomaterial sciences provides an opportunity for the refinement of design and material notions for surgical implants. However, the most important aspect and prime concern is how tissue at the implant site responds to biomechanical disturbances caused by foreign materials.

The literature revealed that zirconia has certain characteristics that make it an excellent material for implants, including biocompatibility and osseointegration which depicts positive soft tissue response with low plaque affinity as well as aesthetics owing to light transmission and color. Additionally, this review discusses the current challenges and prospects of zirconia in dental implantology as well as aims to provide dental professionals and researchers with a comprehensive understanding of zirconia’s potential as a biomaterial in dental implantology. The present overview of available literature intends to highlight and explore the biological characteristics of zirconia for applications in dental implantology. However, research is urgently required to fill in gaps over time for clinical assessments of all zirconia implants, consequently, the implementation of hybrid systems (a titanium screw with a zirconia collar) has recently been suggested.

## Introduction and background

Dental implants have enhanced the quality of life of numerous individuals. Due to their ability to osseointegrate [1] and affordability compared to titanium implants, zirconia dental implants have become more prevalent recently with additional advantageous qualities, such as transparency and white hues, which replicate natural teeth [2]. As it is radiopaque similar to titanium, it is easily visualized on radiographs. When compared to titanium, zirconia is observed to have less bacterial proliferation [3]. Zircon (ZrSiO_4_), which may be found in ores such as zircon and baddeleyite, is widely recognized as a valuable gem [1]. Zirconia is an intriguing material in biological sciences because of its distinctive features such as high strength, hardness, wear resistance, corrosion resistance, an elastic modulus similar to that of steel, a thermal expansion coefficient equivalent to iron, and increased fracture toughness on a mechanical and chemical level [1]. Commercially, there are nine zirconia dental implant systems. Zirconia was first used in dental prosthesis surgery employing endosseous implants in the early 1990s [4]. The tissues around the implant must adhere firmly and securely for dental implants to be successfully incorporated. There is an emerging era in dental implantology that seeks to develop innovative ceramic-based implants with higher periointegration ability, such as osseointegration.

As an alternative to monolithic titanium implants, osseointegration decreases plaque buildup improving the management of soft tissues and aesthetic concerns [3]. This review seeks to highlight and discuss the periointegration perspective of zirconia’s characteristics for the advancement of clinical research using this high-value material, compare them with titanium dental implants, and provide information on zirconia dental implant osseointegration.

## Review

Search methodology

**Figure 1:**
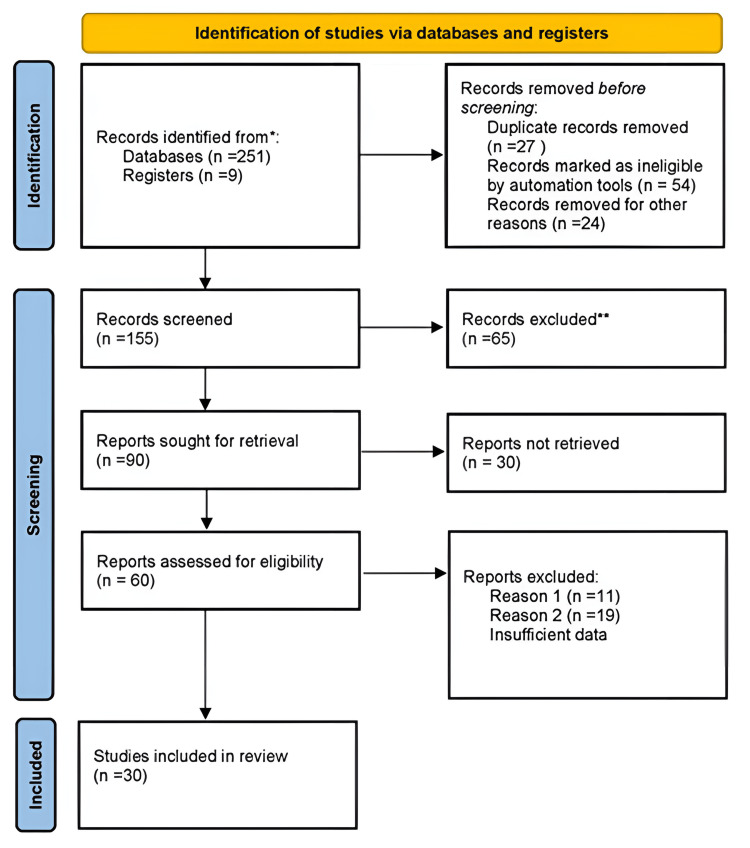
Preferred Reporting Items for Systematic Reviews and Meta-Analyses flowchart describing the selection process.

We conducted a review through PubMed and Google Scholar in August 2022 using keywords such as “Zirconia” (title/abstract), biocompatibility, osseointegration, periointegration, bacterial colonization, and titanium. We additionally explored thoroughly key references from bibliographies of the relevant studies. The search was updated in April 2023. The reviewer monitored and observed the retrieved studies against the inclusion and exclusion criteria based on the title and abstract and then on full texts. For inclusion, both published and unpublished studies in the English language were considered. We excluded studies published in other languages because of resource limitations and full-text articles were unavailable to the reviewer. Figure 1 shows the Preferred Reporting Items for Systematic Reviews and Meta-Analyses flowchart and describes the selection process.

Impact of zirconia’s surface morphology on bone integration

ZrO_2_ surface properties affect implant efficacy and long-term survival. Implant microstructure, surface composition and qualities, and design elements are some of these features [5]. A biomaterial’s material composition along with surface topography is crucial for osseointegration. There are three general types of surface modification techniques, namely, physical such as sandblasting plasma spraying, ion implantation, laser treatment, and pulsed magnetron sputtering; chemical such as acid etching, anodizing, and micro-arc oxidation; and biological such as protein absorption and ion interaction [6]. According to reports, ZrO_2_ nanoparticles are much more biocompatible compared to other nanomaterials including iron oxide, titanium dioxide, and zinc oxide (ZnO). Other investigations have revealed that ZrO_2_ nanoparticles cause barely any cytotoxic effects, although one of the studies demonstrated modest cytotoxic potential, which is consistent with our findings [7]. Some scientists employed zirconia TiO_2_, a conventional nanomaterial with comparable physical and chemical characteristics, as a control group. To monitor osteoblast activity, oxidative stress, and cell shape, as well as the response of osteogenesis, titanium dioxide as well as zirconium dioxide nanoparticles were grown with osteoblastic cell MC3T3-E1 [7].

Mechanical properties of zirconia implants

Zirconia implants have garnered considerable attention in the field of dental implantology due to their unique combination of mechanical strength. Tetragonal zirconia polycrystalline (YTZP) materials along with yttria stabilization have remarkable corrosion resistance along with wear resistance in addition to high flexural strength contrary to other dental ceramics [8]. When the compressive load capacity of zirconia implants based on blade type was evaluated, it was discovered to be sufficient for occlusion [9]. Zirconia’s unloaded fracture strength (512.9 N) was noted to be greater compared to its loaded fracture strength (401.7 N) [10]. The metastable tetragonal structure may be maintained at normal temperature by alloying unadulterated zirconia along with stabilizing oxides such as calcium oxide, magnesium oxide, and yttrium oxide [10].

Fracture Toughness and Resistance to Cracking

This section defines fracture toughness as well as seeks to elucidate its significance in assessing the ability of zirconia implants to withstand crack propagation. It discusses the critical stress intensity factor as a key parameter for characterizing fracture toughness as zirconia has attained immense popularity in dental implantology due to its excellent mechanical properties and biocompatibility. Various factors influence the fracture toughness of zirconia, including grain size, phase composition, sintering conditions, and surface defects. The review explores the crack resistance mechanisms in zirconia implants, including transformation toughening, grain boundary engineering, and stress-induced phase transformations, as well as how these mechanisms enhance the implant’s ability to resist crack initiation and propagation. In addition to this, surface modifications, such as machining, polishing, and glazing, can influence the fracture toughness and crack resistance of zirconia implants. Clinical implications of fracture toughness and crack resistance in zirconia implants play a significant role in preventing implant failure, improving long-term stability, and reducing the risk of complications through testing and measurement techniques to assess fracture toughness and crack resistance in zirconia biomaterials, including indentation-based methods and standardized testing protocols. Acknowledging the significance of these properties and their modulation through material design and processing is essential for dental professionals and researchers seeking to maximize the clinical performance of zirconia implants [8].

Fatigue Behavior and Longevity

This review delves into the crucial aspects of fatigue behavior and longevity of zirconia implants and explores the principles of fatigue, the factors influencing fatigue resistance, and their impact on the long-term performance of zirconia in implants. Factors influencing fatigue resistance of zirconia include material properties, implant design, surface finish, loading conditions, and the oral environment. The mechanisms underlying fatigue failure in zirconia implants include the role of microcracks, stress concentrations, and cyclic loading-induced phase transformations. Aging and degradation processes can affect the longevity of zirconia implants and the impact of environmental factors, such as temperature as well as humidity, on zirconia’s mechanical properties and fatigue resistance over time, and surface modifications impact fatigue resistance and long-term performance. Fatigue behavior along with the longevity of zirconia implants are crucial for establishing and verifying their sustained success in dental implantology. Dental professionals must recognize the significance of these properties and their modulation through material design, surface modifications, and clinical considerations to maximize long-term performance [10].

Biocompatibility of zirconia implants

The biocompatibility of zirconia was examined using a variety of in vitro experiments on osteoblasts, fibroblasts, lymphocytes, and monocytes, along with macrophages. Because zirconia has no immunosuppressed cytotoxic effects on osteoblasts, the cells can elaborate the extracellular matrix by synthesizing a wide range of various essential and structural proteins. According to Liagre et al. [11], zirconia does not cause any inflammatory pathways to arise. Zirconia is biocompatible because it does not possess a pseudo-teratogenic effect [12]. Zirconia underwent biocompatibility studies in vivo as well, and it was observed that after being implanted in soft tissue, it established a thin coating identical to the layer of fibrous tissue in the instance with alumina [13]. Zirconia wear products did not trigger any cytotoxicity in the soft tissue either. The results of a study that implanted pellets of solidified zirconia with 6% Y_2_O_3_ into monkey femurs revealed that zirconia is biocompatible with hard tissue when evaluated in vivo [14]. The proliferation of cells near zirconia was shown to be equivalent to titanium in the study by Kohal et al., although zirconia’s surface modification was found to be more effective and failed to demonstrate osseointegration improvement [15].

Zirconia and its periointegrative properties

Both bone integration and soft tissue integration are connoted by the concept of periointegration. For an implant to be effective and survive for an extended period, both integrations are equally necessary. Both depend on local as well as systemic factors, including the biomaterial’s physicochemical and structural attributes, the tissue (bone tissue and gingival) properties, the placement of implants, and the effectiveness of surgical procedures [16].

Processing methods for zirconia biomaterials

Zirconia, a biocompatible ceramic material, has become a prominent choice for dental and medical applications due to its exceptional properties and performance. The success of zirconia biomaterials in various clinical applications largely depends on the manufacturing processes employed [17]. This review seeks to elucidate a comprehensive analysis of various processing methods employed in the fabrication of zirconia biomaterials for dental implants. It explores the fundamental approach associated with zirconia processing, including powder synthesis, shaping, sintering, and surface modification. The role of processing methods in optimizing the performance of zirconia implants and their potential for enhancing patient outcomes aims to offer valuable insights through an in-depth examination of the latest research, advancements, as well and clinical applications [17].

Conventional and Powder Processing

Conventional powder processing involves transforming raw zirconia powder into a solid, dense structure using a series of steps. These steps may include powder shaping, green machining, sintering, and post-processing. These techniques help control the size, shape, and homogeneity of the zirconia particles, which significantly impacts the final material’s properties [18].

Powder Shaping

After synthesis, the zirconia powder is shaped into the desired form using uniaxial or isostatic pressing. Uniaxial pressing compacts the powder into a mold with a single direction of pressure, while isostatic pressing applies equal pressure from all directions. The valuable insights of this technique in optimizing zirconia biomaterials for betterment and intensified patient care. Various techniques such as uniaxial pressing, isostatic pressing, and recent advancements and innovative methods such as freeze granulation affect powder density, compaction behavior, and green body formation. This property plays a crucial role in determining the initial properties of zirconia and examines how different shaping techniques influence zirconia properties such as density, homogeneity, and microstructure with their implications for eventual processing steps [19].

Green Machining

The shaped green bodies undergo machining to achieve precise dimensions and surface finish. Green machining allows the formation of complex geometries and customization for specific dental implant designs. Precision shaping of zirconia green bodies before sintering is taken care of in the green machining procedure and subsequently delves into the methods and tools used in green machining, including CNC milling and grinding, with their advantages in achieving complex implant geometries and tolerances [19].

Sintering

Sintering is a critical step in conventional processing influencing material density and mechanical properties. The green bodies are heated to a high temperature, which facilitates particle diffusion and neck formation, resulting in densification and enhanced mechanical properties. This section examines conventional and advanced sintering techniques, in conjunction with pressureless sintering, spark plasma sintering, and microwave sintering, along with their effects on zirconia implant properties [20].

Post-processing 

Following sintering, additional treatments such as polishing, glazing, or surface modifications may be applied to improve aesthetics and optimize the biocompatibility of the material [20].

Innovative computer-aided design and computer-aided manufacturing techniques

Advancements in computer-aided design and computer-aided manufacturing (CAD/CAM) have revolutionized the fabrication of zirconia biomaterials, providing enhanced precision and efficiency. CAD/CAM technologies have offered precise and patient-specific solutions. They have simultaneously revolutionized implantology with the latest advancements in digital workflows, software, and hardware, highlighting their impression on zirconia implant design, manufacturing, and clinical outcomes. Cumulative case studies and emerging trends have analyzed the transformative role of innovative contemporary CAD/CAM techniques in optimizing zirconia implants for enhanced patient care [21].

Digital Scanning

In CAD/CAM systems, digital scanners create high-resolution 3D models of patients' dental structures, facilitating accurate customizations and implant planning and introducing the advancements in digital impression techniques, intraoral scanners, and their role in capturing precise data for designing zirconia implant restorations which signifies exploring their innovative applications in zirconia biomaterials and substantiate the significance of CAD/CAM techniques in implantology [22].

Computer-Aided Design

CAD software allows dental professionals to design patient-specific zirconia implants and restorations, tailoring them to match the individual’s anatomy and requirements. CAD/CAM techniques expedite and facilitate zirconia’s customization abutments and restorations, optimizing fit and esthetics and highlighting the advantage of patient-specific prosthetics [22].

Computer-Aided Manufacturing

The CAM process utilizes milling or 3D-printing techniques to produce zirconia-based dental components directly from digital designs. Milling machines employ diamond-coated tools to carve out the zirconia blocks, while 3D printing involves layer-by-layer additive manufacturing. Ingenious 3D printing techniques enable the fabrication of zirconia implant components with unprecedented precision. Future directions and challenges outline potential future directions, including artificial intelligence (AI)-driven design, bioprinting, and teledentistry and provocation accompanying with adoption and advancing CAD/CAM technologies in zirconia implantology [22].

High-Speed Sintering

An emerging CAM method, high-speed sintering (HSS) uses a combination of inkjet printing and rapid sintering to produce fully dense zirconia components in significantly reduced timeframes. To revolutionize the manufacturing of zirconia biomaterials for dental implantology for enhanced patient care HSS has emerged as a cutting-edge manufacturing technology [19]. The principle of HSS is to achieve rapid and precise sintering of zirconia materials and offers several advantages such as reduced processing times, the ability to produce complex geometries, and energy efficiency that states the benefits of HSS in the context of zirconia implant manufacturing. HSS acknowledges well-structured fabrication of patient-specific zirconia implants and customization and enables the production of customized implant solutions [19].

Conventional Sintering

Traditional sintering is typically performed in a high-temperature furnace under controlled conditions to achieve adequate particle bonding and densification. Microwave sintering is an innovative sintering method that utilizes microwave energy to achieve rapid and uniform heating, reducing processing time and energy consumption [23].

Hot Isostatic Pressing

Hot isostatic pressing (HIP) is a post-sintering treatment that involves applying high pressure and temperature to further enhance the material’s density and mechanical properties. HIP enhances the reliability and quality of zirconia implants and its potential for improving patient outcomes to achieve densification and eliminate defects in zirconia materials [19]. HIP-induced microstructural modifications influence zirconia’s behavior, including grain size, phase transformations, and porosity reduction, and have been linked to improved biocompatibility of zirconia. The application of bioactive coatings to improve zirconia implant surface properties delves into how HIP can integrate surface modifications. To underscore the advantages of HIP comparative examination between HIP and conventional zirconia processing techniques, such as uniaxial pressing and isostatic pressing without heating, has been presented [19].

Glazing and Surface Modifications

Applying glaze or surface treatments to zirconia restorations enhances aesthetics, reduces wear on opposing dentition, and optimizes soft tissue response. For understanding the significance and principles of glazing zirconia surfaces, the composition of glaze materials, firing techniques, and the resulting changes in the implant’s surface properties are important. Surface modifications play a pivotal role in optimizing zirconia biomaterials for dental implantology and significantly influence the roughness and topography of zirconia implants and explores how glazing makes a difference to these surface characteristics and its inference for soft tissue attachment as well as bacterial adhesion. The impact of glazing on zirconia’s biocompatibility plays a pivotal role in promoting osseointegration demonstrating improved bone response [24].

Clinical applications of zirconia in dental implantology

Dental implantology has undergone significant advancements, and zirconia has played a pivotal role in revolutionizing restorative dental procedures such as single-tooth zirconia implants which have gained considerable attention for single-tooth replacements due to their excellent biocompatibility, mechanical strength, and aesthetic appeal. Moreover, partial and full arch implant-supported prostheses offer a durable and aesthetically pleasing solution for restoring multiple missing teeth. In addition to this, zirconia abutments and restorations provide an alternative to conventional metal-based restorations and discuss the advantages of zirconia abutments, including reduced soft tissue discoloration and improved gingival health, as well as evaluate their clinical performance [24].

Overcoming challenges in complex cases

Complex implant cases, such as limited bone volume and angulated implants, often require advanced solutions such as zirconia implant-supported overdentures which offer a secure and cost-effective treatment option for edentulous patients and explore clinical feasibility, advantages, and limitations of using zirconia for implant-retained overdentures. Complex cases often involve patients with limited bone volume, which needs bone augmentation procedures, explore the challenges of bone augmentation with zirconia implants, and discuss advancements such as guided bone regeneration techniques along with customized implant designs to optimize implant placement in compromised bone conditions.

Future perspectives and advancements

Advancements in zirconia materials, manufacturing techniques, and surface modifications present exciting opportunities for further improving clinical outcomes. This section explores potential future developments and their implications for zirconia’s clinical applications in dental implantology [25]. Recent research has emphasized the growing significance of zirconia-based nanomaterials and their applications in implantology which have emphasized the development of nanoscale modifications of zirconia which may amplify osseointegration as well as soft tissue integration. In addition to this, bioactive surface modifications explore the latest advancements in zirconia surface treatments, such as bioglass and hydroxyapatite coatings, and their potential clinical impact. 3D printing and customization are revolutionizing and offering unparalleled customization for the precise fabrication of patient-specific zirconia implants and restorations [26]. In optimizing zirconia implant placement, advancements in multimodal imaging, including cone-beam computed tomography along with intraoral scanning, are enhancing the accuracy of treatment planning and guided surgery. Some hybrid materials such as a combination of zirconia with other biocompatible substances may offer enhanced properties and versatility [26].

Complications and challenges with zirconia implants

Zirconia implants have demonstrated impressive mechanical strength; however, there is an increased risk of vulnerability to fracture and chipping. Contributing factors of zirconia implant fractures include excessive occlusal forces, inadequate design, and flawed materials. Interaction between zirconia implants and peri-implant soft tissues plays a critical role in implant success [27]. Proper implant-abutment connection design and surface modifications are essential to minimize these complications [28]. Zirconia implants are predominantly used in a cement-retained restoration approach. However, complications related to cementation, such as excess cement remnants, lead to peri-implant inflammation and implant failure. Strategies for mitigating cementation-related complications, including the use of retrievable abutments and the development of adhesive techniques, have been explored [29]. While zirconia is generally considered biocompatible, rare cases of allergic reactions to zirconia have been reported [30]. Therefore, proper patient screening and material selection are important to prevent such complications. Zirconia implants can be more expensive than traditional titanium implants, which may present financial challenges for some patients. The cost implications and considerations for zirconia implants are quite challenging [31]. Despite several difficulties, dental implant treatment generally remains a reliable and secure alternative for edentate and partly dentate individuals [32].

Recent advancements and future perspectives

The increasing interest in zirconia for dental implantology and its potential to overcome the limitations of traditional materials emphasizes the need for continuous research and advancements to optimize zirconia’s performance in dental implant treatments. Recent advancements in zirconia manufacturing techniques, including HSS and CAD/CAM, have revolutionized the production of zirconia dental implants which have led to increased precision, efficiency, and customized implant designs [33].

## Conclusions

Zirconia has undoubtedly revolutionized the dental implantology landscape, offering remarkable mechanical properties, biocompatibility, and aesthetic appeal. This review article emphasizes the importance of understanding zirconia’s properties, processing methods, and clinical applications to make informed decisions in dental implantology. Zirconium oxide has several potential applications in dental crowns, post and core systems, and implants. Numerous in vitro and in vivo tests have shown that the material has a high fracture resistance and is suitable for usage in stress-bearing regions. The properties of the zirconium oxide surface such as an absence of micro gaps between the fixtures and the absence of plaque adhesion to the zirconium oxide restrict microbial infiltration. Zirconia has emerged as a versatile as well as promising biomaterial in dental implantology, offering an exceptionally comprehensive understanding of zirconia’s role in dental implantology, despite the challenges and complications that may arise.
